# Active-Site Protonation States in an Acyl-Enzyme Intermediate of a Class A β-Lactamase with a Monobactam Substrate

**DOI:** 10.1128/AAC.01636-16

**Published:** 2016-12-27

**Authors:** Venu Gopal Vandavasi, Patricia S. Langan, Kevin L. Weiss, Jerry M. Parks, Jonathan B. Cooper, Stephan L. Ginell, Leighton Coates

**Affiliations:** a Biology and Soft Matter Division, Oak Ridge National Laboratory, Oak Ridge, Tennessee, USA; b Biosciences Division, Oak Ridge National Laboratory, Oak Ridge, Tennessee, USA; c Birkbeck University of London, London, United Kingdom; d Structural Biology Center, Argonne National Laboratory, Argonne, Illinois, USA

**Keywords:** β-lactamase, aztreonam, acyl-enzyme complex, neutron structure, X-ray structure

## Abstract

The monobactam antibiotic aztreonam is used to treat cystic fibrosis patients with chronic pulmonary infections colonized by Pseudomonas aeruginosa strains expressing CTX-M extended-spectrum β-lactamases. The protonation states of active-site residues that are responsible for hydrolysis have been determined previously for the apo form of a CTX-M β-lactamase but not for a monobactam acyl-enzyme intermediate. Here we used neutron and high-resolution X-ray crystallography to probe the mechanism by which CTX-M extended-spectrum β-lactamases hydrolyze monobactam antibiotics. In these first reported structures of a class A β-lactamase in an acyl-enzyme complex with aztreonam, we directly observed most of the hydrogen atoms (as deuterium) within the active site. Although Lys 234 is fully protonated in the acyl intermediate, we found that Lys 73 is neutral. These findings are consistent with Lys 73 being able to serve as a general base during the acylation part of the catalytic mechanism, as previously proposed.

## INTRODUCTION

β-lactam antibiotics inhibit bacterial cell wall biosynthesis by targeting penicillin-binding proteins (PBPs). The binding of β-lactam antibiotics to PBPs renders them chemically inert, causing bacterial cell death. To counter such powerful antimicrobials, bacteria have evolved to produce β-lactamase enzymes, which cleave the amide bond within the β-lactam ring via a general base hydrolysis mechanism (1–3). According to the Ambler classification (4), β-lactamases can be divided into four distinct groups (classes A to D). Classes A, C, and D consist of all serine-reactive hydrolases, whereas the class B enzymes are metalloenzymes that use a Zn^2+^-bound water molecule to hydrolyze the amide bond of the β-lactam ring. Extended-spectrum β-lactamases (ESBLs) arose in the 1980s and exhibit increased hydrolytic activity against the first-, second-, and third-generation extended-spectrum cephalosporins and monobactams (3–6). Toho-1 β-lactamase, also classified as CTX-M-44, is a class A ESBL. Like most other class A β-lactamases, it comprises two highly conserved domains (α/β and α), with the active site located at the interface of these two domains (7, 8).

**FIG 1:**
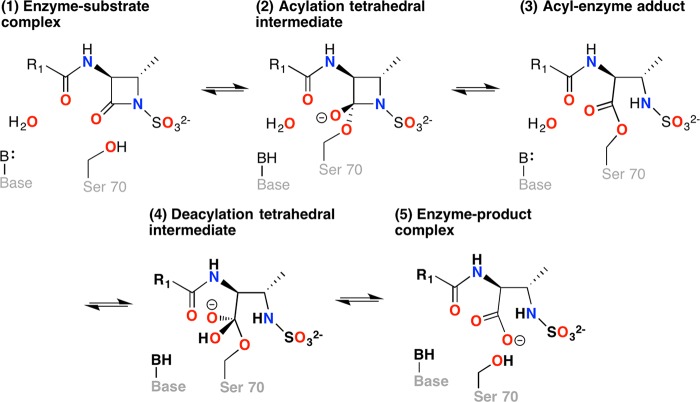
Catalytic cycle of a class A β-lactamase illustrated for a monobactam substrate. All class A β-lactamases employ an active site serine nucleophile to cleave the β-lactam bond of the substrate in a two-step acylation-deacylation reaction cycle that leads to overall hydrolysis. The acylation reaction initiates with the formation of a precovalent substrate complex (stage 1). A general base-catalyzed nucleophilic attack on the β-lactam carbonyl by the serine hydroxyl proceeds through a tetrahedral intermediate (stage 2) to form a transient acyl-enzyme adduct (stage 3). In the deacylation step, the acyl-enzyme adduct (stage 3) undergoes a general base-catalyzed attack by a hydrolytic water molecule to form a second tetrahedral intermediate (stage 4), which then collapses to form a postcovalent product complex (stage 5), from which the hydrolyzed product is released.

In contrast to most other β-lactams, which have at least two rings, in monobactams the β-lactam ring is not fused to another ring. Aztreonam has a large R group attached to the β-lactam ring that inhibits its hydrolysis by Toho-1 β-lactamase (9). The proposed catalytic mechanism of monobactam breakdown by a class A β-lactamase is shown in Fig. 1. After substrate binding, Ser 70 attacks the carbonyl carbon of the β-lactam ring to form an acyl-enzyme intermediate, which is then deacylated to liberate the inactivated antibiotic (1, 2). Glu 166 plays a vital role in the deacylation step (Fig. 1, stages 3 to 5), where it acts as the activating base of a hydrolytic water molecule (10, 11). Mutating Glu 166 halts the reaction at the acyl intermediate (stage 3), allowing this state to be characterized structurally. Glu 166 has also been proposed to act as the catalytic base in the acylation step of the reaction (Fig. 1, stages 1 to 3) in which this residue deprotonates the hydroxyl of Ser 70 via a water molecule before Ser 70 attacks the carbonyl carbon of the β-lactam ring (8, 12, 13). Wild-type β-lactamases rapidly hydrolyze β-lactam antibiotics, making it virtually impossible to trap the acyl-enzyme intermediate.

It has been observed that Glu 166 mutants are still able to form acyl-enzyme intermediates, albeit with rate decreases of between 100-fold and 1,000-fold (11, 14, 15). This observation strongly suggests that Lys 73 can act as general base during the acylation step, as proposed in a number of studies (10, 11, 16, 17). Lys 73 is highly conserved throughout the serine-reactive β-lactamase families as well as the penicillin-binding proteins (18). This active-site residue is in close proximity to other catalytic residues, including Ser 70, Ser 130, and Glu 166. Mutation of Lys 73 to Arg results in a 100-fold decrease in acylation activity (19), indicating that Lys 73 participates in catalysis, although its role is still unclear. However, when both Lys 73 and Glu 166 are mutated, the rate constants for the acylation reaction decrease by 10,000-fold (19).

High-resolution X-ray structures have been determined previously for an Arg 274 Asn/Arg 276 Asn double mutant and a Glu 166 Ala/Arg 274 Asn/Arg 276 Asn triple mutant of Toho-1 β-lactamase in its apo form (20). The mutations Arg 274 Asn and Arg 276 Asn prevent crystal twinning and increase diffraction resolution (20) without dramatically affecting the kinetics of the enzyme (9). Neutron crystal structures have also been determined for both of these variants, clearly revealing that Lys 73 and Lys 234 are fully protonated and Glu 166 is anionic in the apoenzyme (21, 22). However, the protonation states of active-site residues in a monobactam acyl-enzyme intermediate have not yet been determined.

To characterize β-lactam hydrolysis in a class A β-lactamase, we determined both neutron and high-resolution 15 K X-ray acyl-intermediate structures of the Toho-1 Glu 166 Ala/Arg 274 Asn/Arg 276 Asn mutant in complex with the monobactam antibiotic aztreonam, which is often used to treat bacterial lung infections in patients with cystic fibrosis (23). The aztreonam acyl intermediate was trapped for combined neutron and X-ray structure determination by mutating Glu 166 to Ala.

## RESULTS AND DISCUSSION

**FIG 2:**
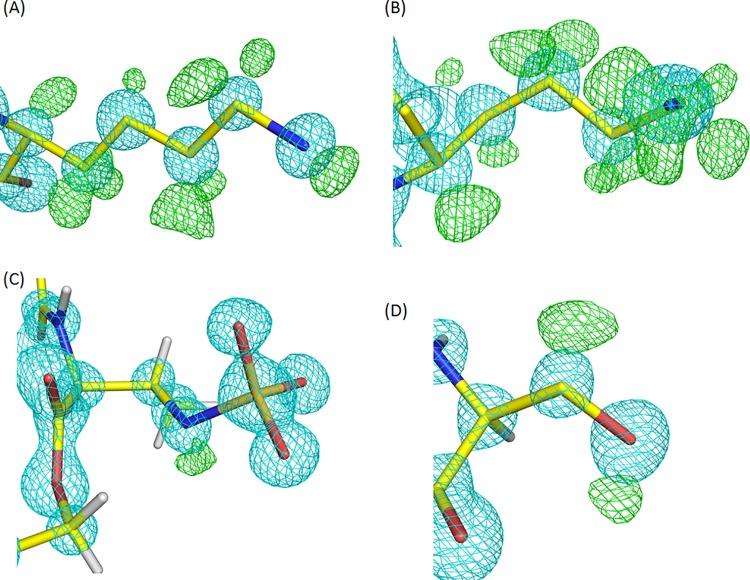
15 K X-ray electron density for key residues in the active site. The 2F_o_-F_c_ density at 3.7 σ is shown in cyan, and the F_o_-F_c_ density at +2.5 σ is shown in green. Side-chain deuterium atoms have been omitted from (A) Lys 73, (B) Lys 234, (C) aztreonam, and (D) Ser 130 to elucidate their actual positions.

**FIG 3:**
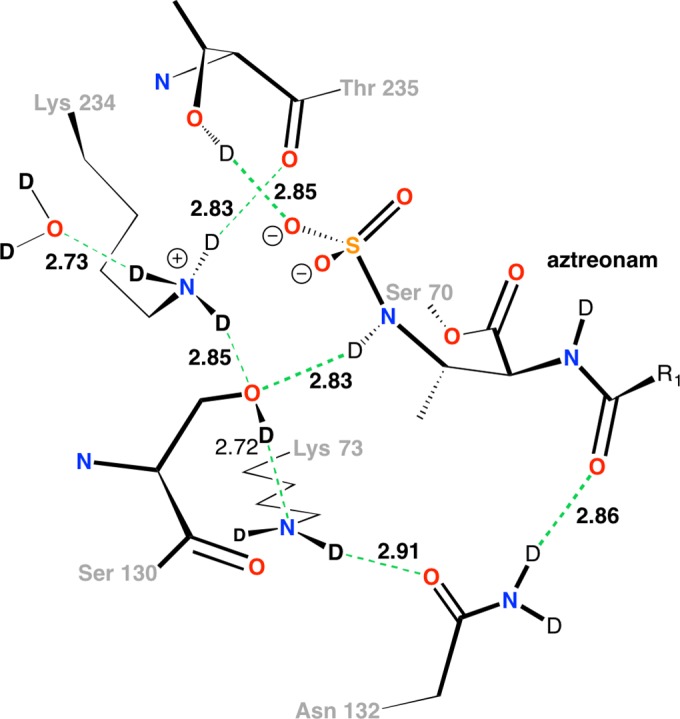
Hydrogen bonding network in the active site. All distances (between donor and acceptor heavy atoms [indicated in angstroms]) are from the 15 K acyl-enzyme intermediate X-ray structure.

The combination of neutron diffraction and 15 K X-ray diffraction enabled us to visualize most of the deuterium atoms within the active site of the monobactam antibiotic binding site. As a result, we probed the protonation states and hydrogen-bonding pattern therein, which allowed us to infer key aspects of the hydrolysis mechanism. In the 15 K X-ray structure, the average atomic displacement parameter (ADP) or B factor for protein main chain atoms is 7.68 Å^2^, with an average of 12.92 Å^2^ for protein side chain atoms. However, at around 5 Å^2^, the values for the main-chain and side-chain ADPs of atoms in the three active site key residues, Lys 73, Ser 130, and Lys 234, are considerably lower than the average. Thus, we observed clear omit electron density for almost all of the deuterium atoms within the active site. The side chain of Lys 73 shows omit electron density for most of the deuterium atoms on the side chain (Fig. 2A). Omit electron density is present for both of the deuterium atoms on the C_β_, C_δ_, and C_ε_ atoms, but only a single deuterium is visible on C_γ_. The omit electron density around N_ζ_ of Lys 73 is not as clear as on the rest of the side chain but seems to indicate that a hydrogen bond is formed with O_δ_ of Asn 132 (2.90 Å). It also suggests the presence of a hydrogen bond (2.90 Å) with the main-chain carbonyl oxygen of Ser 130. The omit electron density for the side chain of Lys 234 confirms that it is in the ND3+ cationic form and reveals the positions of all three deuterium atoms (Fig. 2B). It forms hydrogen bonds with O_γ_ of Ser 130 (2.84 Å), the main-chain carbonyl oxygen of Thr 235 (2.83 Å), and a nearby water molecule (2.73 Å). The omit electron density around the β-lactam ring nitrogen of aztreonam clearly indicates that it is protonated, with the deuterium orientated toward O_γ_ of Ser 130, with which it forms a hydrogen bond (2.84 Å) (Fig. 2C). The omit electron density for D_γ_ of Ser 130 indicates that it is orientated toward N_ζ_ of Lys 73 (Fig. 2D), with which it forms a hydrogen bond (2.72 Å). The inferred details of these interactions within the active site are shown in Fig. 3. Several other interactions between the monobactam and the protein are also present. The main chain carbonyl of Ser 237 hydrogen bonds to an amide group of the monobactam (2.77 Å), while one of the dual conformations of the side chain of Ser 237 interacts with a carboxylate group on the monobactam (2.70 Å). The carbonyl group of the monobactam also forms interactions with the amino groups of Asn 104 (3.00 Å) and Asn 132 (2.87 Å).

**FIG 4:**
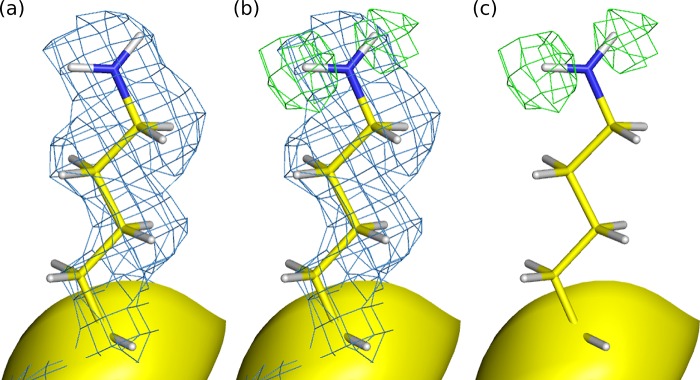
The neutron data uniquely reveal that Lys 73 is in the neutral ND_2_ protonation state. (a) The 2F_o_-F_c_ 293 K nuclear density map (blue mesh) is contoured at 1.5 σ. (b) The 2F_o_-F_c_ 293 K nuclear density map (blue mesh) is contoured at 1.5 σ, and the deuterium omit difference density map (green mesh) is contoured at 1.5 σ for the left deuterium atom and +1.2 σ for the right deuterium atom. (c) The deuterium omit difference density map (green mesh) is contoured as described for panel b.

The 293 K neutron diffraction data are very similar to the 15 K X-ray structure data in that Lys 234 is present in the ND_3_^+^ form and the D_γ_ atom of Ser 130 is oriented toward Lys 73. However, in deuterium omit maps, the neutron structure clearly shows that Lys 73 is in the neutral ND_2_ form (Fig. 4) and is hydrogen bonded to O_δ_ of Asn 132.

Several investigators have proposed roles for both Lys 73 and Ser 130 in the protonation of the β-lactam ring nitrogen (11, 14, 24–26). The closest proton source to the β-lactam nitrogen is the hydroxyl group of Ser 130, but until now it has not been clear whether Lys 234 or Lys 73 acts as the proton source to complete the acylation step in the catalytic mechanism. The observed protonation states of Lys 234 (–ND_3_^+^) and Lys 73 (–ND_2_) from our neutron and X-ray structures indicate that Lys 73 is the proton source, as previously suggested (11, 14, 27). In the Glu 166 Ala mutant, the Lys 73 side chain most likely acts as a general base in the acylation reaction, having extracted a proton from Ser 70. A second proton transfer pathway would initiate the transfer of two protons. The first proton would be transferred from N_ζ_ of Lys 73 to O_γ_ of Ser 130, and the second proton would be transferred from O_γ_ of Ser 130 to the β-lactam nitrogen, thereby leaving Lys 73 in the –ND_2_ neutral form observed in our X-ray and neutron structures. The ability of Glu 166 Ala mutants to form acyl-enzyme intermediates indicates that Lys 73 is able to act as a general base in the acylation part of the reaction, as supported by enzyme kinetics measurements (15, 28). However, the large drop in the acylation rate of the Glu 166 Ala mutant clearly shows that Glu 166 takes part in the acylation step in which it has also been proposed to act as the general base (12, 13, 29). On the basis of quantum mechanical/molecular mechanical (QM/MM) calculations, the Glu 166 Ala mutation was proposed to lower the pK_a_ of Lys 73, enabling it to act as a general base during the acylation step (14, 17). Our structures show direct (and indirect) evidence that the mutation of Glu 166 to Ala alters the pK_a_ of Lys 73, possibly reverting the enzyme to a penicillin-binding protein capable of binding β-lactams but incapable of releasing them. The presence of an acylated aztreonam within the active site is also likely to affect the pK_a_ values of the catalytic residues.

Previous studies by Gibson et al. (19) have shown that the Lys 73 Arg mutant shows a 100-fold lower rate for the acylation reaction and that a dual Lys 73 Arg/Glu 166 Asp mutant shows a 10,000-fold lower rate. Thus, the dual presence and interaction of both Glu 166 and Lys 73 are vital in the acylation mechanism of the class A β-lactamase enzymes for which both amino acids are required for catalytic competency.

### Conclusions.

Using both X-ray and neutron crystallography, we have located the positions of most of the hydrogen (deuterium) atoms within the active site of Toho-1 β-lactamase in its acyl-enzyme intermediate state in complex with aztreonam. These structures suggest that Lys 73 can act as a general base in the acylation step of the β-lactam hydrolysis reaction, as has been previously proposed (11, 14, 17, 30). The experimentally observed deuterium positions seen in our neutron and X-ray structures are a match to those put forward by Mobashery and coworkers from their studies on TEM-1 β-lactamase in which they used different techniques (QM/MM and nuclear magnetic resonance [NMR]) with a different substrate and yet determined the same protonation states (14, 17). This indicates that these results, which have been verified by four different techniques, are broadly applicable to class A β-lactamase catalysis.

## MATERIALS AND METHODS

Perdeuteration is the complete replacement of hydrogen with deuterium, which dramatically increases the signal-to-noise ratio of neutron diffraction data while also enabling the experimental location of all the atoms in the protein (21, 22, 31). Using perdeuterated protein crystals, neutron diffraction data on the Glu 166 Ala/Arg 274 Asn/Arg 276 Asn Toho-1 β-lactamase aztreonam acyl-enzyme intermediate were collected on the macromolecular neutron diffractometer (MaNDi) beamline at the Spallation Neutron Source (32, 33). A perdeuterated protein crystal roughly 0.9 mm^3^ in volume was placed for 2 to 3 h in a reservoir D_2_O solution containing 2.7 M ammonium sulfate, 0.1 M sodium citrate (pH 6.1), and 5.0 mM aztreonam. The crystal was then sealed in a quartz capillary for neutron data collection on MaNDi using neutrons with wavelengths between 2 to 4 Å to collect a series of time of flight wavelength-resolved Laue diffraction images. A total of 10 wavelength-resolved Laue diffraction images were collected with a 6-h exposure time per image. Between images, the sample was rotated by 10 degrees on the ϕ axis. In addition to neutron diffraction data, we also collected a 15 K X-ray diffraction data set on a smaller perdeuterated protein crystal to beyond atomic resolution (1.10 Å) on the SBC-CAT sector 19 beamline at the Advanced Photon Source (APS). High-resolution monochromatic (0.67-Å) X-ray diffraction data were collected on the acyl-enzyme complex using a Cryo Industries of America Cryocool helium cryostream at 15 K. The 15 K X-ray data were processed using the XDS (34) package, whereas the neutron data were processed using the Mantid package (35) and the Lauenorm program from the Lauegen package (36). Lauenorm was then used for wavelength normalization of the Laue data and scaling between Laue diffraction images. The PHENIX suite (37) was used to refine the 293 K neutron and 15 K X-ray data to convergence separately. Further refinement was done on the neutron data using Shelx (38), and all model building was done using the Coot (39) molecular graphics program. The data reduction and refinement statistics for the two structures are given in File S1 of the supplemental material.

### Accession number(s).

Experimental data and coordinates were deposited with PDB identifiers 5G18 and 5KSC.

## Supplementary Material

Supplemental material
