# Supplementary material for: Active-Site Protonation States in an Acyl-Enzyme Intermediate of a Class A β-Lactamase with a Monobactam Substrate
Source: Antimicrob Agents Chemother. 2016 Dec 27;61(1):e01636-16. doi: 10.1128/AAC.01636-16 (PMC5192116; doi:10.1128/AAC.01636-16)
Supplement: Supplemental material [file supp_61_1_e01636-16__index.html]

Active-Site Protonation States in an Acyl-Enzyme Intermediate of a Class A β-Lactamase with a Monobactam Substrate — Supplemental material 

# Active-Site Protonation States in an Acyl-Enzyme Intermediate of a Class A β-Lactamase with a Monobactam Substrate

## Supplemental material

- Supplemental file 1 -

  File S1: expression, purification, and crystallization details; data collection and refinement tables.

  PDF, 198K
