# Supplementary material for: Active-Site Protonation States in an Acyl-Enzyme Intermediate of a Class A β-Lactamase with a Monobactam Substrate
Source: Antimicrob Agents Chemother. 2016 Dec 27;61(1):e01636-16. doi: 10.1128/AAC.01636-16 (PMC5192116; doi:10.1128/AAC.01636-16)
Supplement: Supplemental material [file AAC.01636-16_zac001175806s1.pdf]

Supporting information Section

Expression, Purification, and Crystallization of pd-Toho-1 E166A/R274N/R276N

The perdeuterated enzyme was purified and crystallized as described previously. Briefly, using an *Escherichia coli*-based expression system, the pd-Toho-1 E166A/R274N/R276N was expressed to a high yield in a fully deuterated minimal medium using a fed-batch fermentation protocol. Large crystals for neutron diffraction, along with smaller crystals suitable for X-ray diffraction, were grown at 20 °C via the batch crystallization method using 300 µl of a 10 mg/ml protein concentration in a solution containing 2.0 M ammonium sulfate and 0.1 M sodium citrate (pH 6.1) prepared in D_2O. For ligand soaking crystals were placed for 2-3 h in a reservoir solution containing 2.7 M ammonium sulfate, 0.1 M sodium citrate (pH 6.1), and 5.0 mM aztreonam. The crystals were then mounted either into a capillary for 293 K data collection or placed momentarily in a reservoir solution containing a cryoprotectant (30% w/v perdeuterated trehalose) and then flash-frozen in liquid nitrogen.

Data collection statistics

X-ray Diffraction Data Collection (15 K) and Refinement Statistics

PDB Accession Code	5G18
Unit-cell parameters (Å)	a = 72.45, b = 72.45, c = 97.61<br>α = β = 90° and γ = 120°
Space group	$P3_221$
No. of unique reflections	113,164
Resolution range (Å)	33.96-1.10 (1.16-1.10)
Multiplicity	6.4 (6.4)
I/σ(I)	9.3 (2.4)
R_{merge} (%)	6.6 (35.7)
R_{pim} (%)	2.4 (13.4)
Data completeness (%)	94.1 (95.6)
Crystallographic refinement	
R_{factor} (%)	11.31
R_{free} (%)	13.43
$RMSD_{Bonds}$ (Å)	0.012
$RMSD_{Angles}$ (°)	1.683
Ramachandran plot	
Outliers (%)	0.38

Favored (%)	97.4
Rotamer Outliers (%)	1.36

Highest resolution shell is shown in parentheses

Neutron Diffraction Data Collection (293 K) and Refinement Statistics

PDB Accession Code	5KSC
Unit-cell parameters (Å)	a = 73.30, b = 73.30, c = 90.10
	α = β = 90° and γ = 120°
Space group	$P3_221$
No. of unique reflections	13,173
Resolution range (Å)	14.73-2.10 (2.17-2.10)
Multiplicity	3.1 (2.0)
I/σ(I)	10.1 (4.2)
R_{merge} (%)	16.0 (26.0)
R_{pim} (%)	8.7 (16.1)
Data completeness (%)	71.5 (50.1)
Crystallographic refinement	
R_{factor} (%)	24.25
R_{free} (%)	28.27
Ramachandran plot	
Outliers (%)	0.77
Favored (%)	94.97
Rotamer Outliers (%)	0

Highest resolution shell is shown in parentheses
